# Predicting Agitation Stability of Monoclonal Antibodies during Developability Assessment

**DOI:** 10.1021/acs.molpharmaceut.6c00092

**Published:** 2026-04-24

**Authors:** Michaela Cohrs, Nevena Pagureva, Utku Ozbulak, Wesley De Neve, Kevin Braeckmans, Stefaan De Smedt, Slavka Tcholakova, Zahari Vinarov, Hristo L. Svilenov

**Affiliations:** † Laboratory of General Biochemistry and Physical Pharmacy, 26656 Ghent University, Ottergemsesteenweg 460, 9000 Ghent, Belgium; ‡ Department of Chemical and Pharmaceutical Engineering, Faculty of Chemistry and Pharmacy, Sofia University, 1 J. Bourchier Ave., 1164 Sofia, Bulgaria; § Center for Biosystems and Biotech Data Science, Ghent University, Global Campus, 119-5 Songdomunhwa-ro, Incheon 21985, Republic of Korea; ∥ Department of Electronics and Information Systems, 26656 Ghent University, Technologiepark-Zwijnaarde 126, 9052 Ghent, Belgium; ⊥ Biopharmaceutical Technology, TUM School of Life Sciences, 9184 Technical University of Munich, Emil-Erlenmeyer-Forum 5, 85354 Freising, Germany

**Keywords:** antibody drugs, developability, interfacial stability, biophysical characterization, surface tension, interfacial rheology

## Abstract

Developability assessment facilitates the selection of antibody drug candidates with desirable pharmaceutical properties. However, it remains uncertain whether agitation-induced aggregation can be predicted from standard developability parameters. Here, we investigated whether key biophysical parameters predict agitation-induced aggregation of monoclonal antibodies (mAbs). To this end, we generated a benchmark data set by characterizing the aggregation upon agitation in the presence of an air–liquid interface of ten approved mAbs reformulated in a common surfactant-free buffer. The extent of aggregation varied substantially among mAbs and was primarily dependent on antibody identity. Flow imaging microscopy combined with machine learning revealed micrometre-sized aggregates with distinct morphologies, consistent with aggregation at air–liquid interfaces. Examination of thin liquid films and foams confirmed the presence of aggregates directly at the air–liquid interface and, therefore, the critical role of this interface for antibody aggregation during agitation. We then applied fluorescence-based, light scattering, and chromatographic techniques to determine standard developability parameters for each mAb, including apparent melting temperature (*T*_m_), nonreversibility onset temperature (*T*_nr_), aggregation onset temperature (*T*_agg_), diffusion self-interaction parameter (*k*_D_), hydrophobic interaction chromatography retention time, and relative monomer yield after isothermal refolding from chemical denaturants. Notably, none of these parameters correlated with agitation-induced aggregation. Finally, we assessed the surface properties of the mAbs via drop shape analysis and found that the combination of surface pressure and elastic modulus yields a good correlation with the concentration of micrometre-sized aggregates formed due to agitation. Overall, these findings highlight limitations in predicting mAb interfacial stability using standard developability assays and underscore the importance of studying antibody behavior at interfaces.

## Graphical abstract

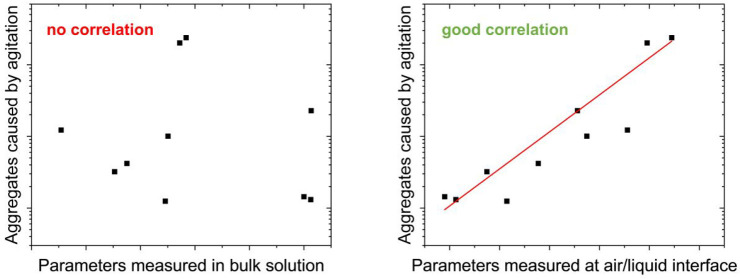

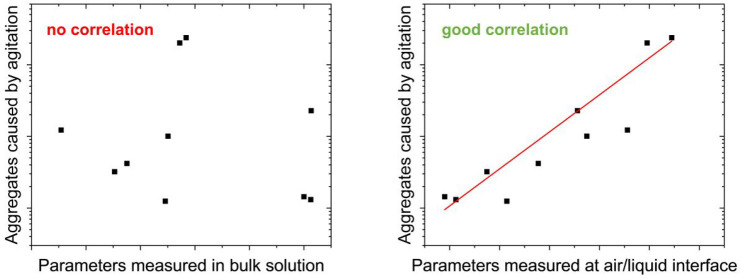

## Introduction

Therapeutic monoclonal antibodies (mAbs) are susceptible to both chemical and physical degradation.^1–4^ For example, adsorption to air–liquid interfaces can lead to antibody unfolding and aggregation, a process that is further exacerbated by agitation due to dynamic interface renewal.^5–7^ Air–liquid interfaces are present throughout the entire lifecycle of an antibody drug, posing significant stability challenges.^8–11^ Although antibody formulations contain stabilizing excipients, approximately 60% of marketed mAbs require compounding before administration, which involves dilution into infusion bags.^12,13^ This dilution reduces the concentration of stabilizers, increasing the risk of degradation. Moreover, infusion bags often contain an air headspace to facilitate complete administration of the bag content.^14^ The headspace creates a large air–liquid interface that can promote mAb aggregation during mechanical stress, such as agitation during in-hospital transport and handling.^10,15–17^

Antibodies are amphiphilic macromolecules that readily adsorb at the air–liquid interface.^6,7,9,18–24^ Upon adsorption, mAbs can form a densely packed amorphous layer, exhibiting rheological properties characteristic of soft glasses.^25^ This layer behaves as a viscoelastic film, with its properties controlled by interprotein interactions that are influenced through antibody identity, stability, conformation, and mobility within the film as well as external factors such as temperature.^6,7,9,18–21,23,24,26^ Film formation is frequently accompanied by structural changes in antibodies, such as unfolding.^19–23^ At low pressure, these films remain compressible, while at high pressures, they become incompressible and prone to disruption.^6,7,9,18–21,23–25,27^ When disrupted, protein aggregates are released into the bulk solution, and new antibody adsorbs to the available interface.^5–7^ This interfacial-induced aggregation pathway reduces the concentration of native antibody monomers and promotes the formation of aggregates of varying sizes, which may trigger immunogenic responses and lead to adverse effects.^8,28–31^

Early identification of mAbs with low aggregation propensity is critical for developing stable biotherapeutics.^32–35^ High inherent interfacial stability can reduce the need for surfactants, which are typically added during formulation to mitigate adsorption and aggregation at interfaces. By reducing the dependence on surfactants, one could avoid issues related to undesired and complex interactions between proteins and surfactants, as well as problems with surfactant quality and degradation.^36–42^

To find antibodies with high stability, developability assessment using fast biophysical assays has become integral to drug candidate screening.^32–35^ Thermal assays such as differential scanning fluorimetry (DSF) are well established for estimating the conformational stability by determining the apparent melting temperature (*T*_m_). Dynamic light scattering (DLS) combined with a heat ramp is used to measure the aggregation onset temperature (*T*_agg_).^32,43–46^ Recent advances introduced additional descriptors in developability assessment, including the nonreversibility onset temperature (*T*_nr_), accessible via modulated scanning fluorimetry (MSF).^43,47,48^ In addition, isothermal assays provide important complementary parameters.^49^ For example, colloidal stability can be quantified through the diffusion self-interaction parameter (*k*_D_) determined by DLS. Hydrophobic interaction chromatography (HIC) can be used to assess mAb hydrophobicity.^1,32–34^ Each of these parameters provides an important piece of information, and a combination of several parameters can identify antibody drug candidates with favorable stability.^32,33,35,50,51^ However, these analyses primarily address mAb stability in bulk solution, whereas stability at interfaces can differ significantly.^24,34,52^

In this study, we investigated whether standard developability parameters can predict agitation-induced aggregation of mAbs. Ten approved mAbs were reformulated in a surfactant-free buffer (acetate pH 5, 0.9% NaCl) to mimic conditions typically encountered in infusion bags. Usage of 0.9% NaCl reflects the widespread use of saline as a diluent for mAb products.^12,13^ Saline is slightly acidic, and to maintain this pH, we added 10 mM acetate buffer.^53^ Acetate buffer is also broadly employed in commercial mAb formulations^54^ and is compatible with developability assays.^35,43,55,56^ The mAbs were then subjected to agitation stress, and aggregation was assessed using multiple orthogonal techniques. The largest differences between the mAbs were observed in the concentration of micrometre-sized aggregates. Morphology assessment of aggregates, inspection of thin liquid films and foams, as well as agitation in the absence of air, confirmed the critical role of the air–liquid interface during agitation-induced aggregation. In parallel, we determined standard developability parameters for each mAb, including *T*_m_, *T*_nr_, *T*_agg_, *k*_D_, HIC retention time, and monomer recovery from the ReFOLD assay. Notably, none of these biophysical parameters distinguished antibodies prone to agitation-induced aggregation from those that were stable. In contrast, the combination of surface pressure and elastic modulus, derived from surface tension and surface rheology measurements, exhibits strong predictive power for agitation-induced aggregation. These findings highlight that developability assessment workflows need dedicated methods to predict mAb interfacial stability.

## Materials and Methods

### Proteins and Chemicals

Ten commercial mAb products were obtained from the Ghent University Hospital (Table 1). The excipients from the commercial products were removed via cation-exchange chromatography using a HiTrap SP FF column (cytiva, Marlborough, USA), extensive rinsing, and a NaCl gradient for elution on an ÄKTA pure (cytiva, Marlborough, USA). The eluates were dialyzed extensively against 10 mM sodium acetate buffer, pH 5, with 0.9% NaCl. Successful surfactant removal was confirmed by comparing the surface tension and rheology of purified antibody to a surfactant-free antibody drug substance. All samples were diluted to 0.45 ± 0.05 mg/mL. Samples for biophysical analyses were frozen and stored at −80 °C until thawed and measured. The concentration was determined with UV spectroscopy (see below). All chemicals were of pharma grade or higher. Ultrapure water (Milli-Q, Merck, Darmstadt, Germany) was used.

**Table 1:** List of Commercial Products Used to Purify mAbs for Investigation of Interfacial Stability

**abbreviation**	**INN**	**commercial product**	**format**	**theoretical** **pI** ^a^
BEVA	bevacizumab	Mvasi	IgG1k	8.09
NIVO	nivolumab	Opdivo	IgG4k	7.93
RITU	rituximab	MabThera	IgG1k	8.66
PEMB	pembrolizumab	Keytruda	IgG4k	7.63
DARA	daratumumab	Darzalex	IgG1k	8.26
INFL	infliximab	Remsima	IgG1k	7.35
OCRE	ocrelizumab	Ocrevus	IgG1k	8.49
VEDO	vedolizumab	Entyvio	IgG1k	8.09
TRAS	trastuzumab	Herceptin	IgG1k	8.45
ISAT	isatuximab	Sarclisa	IgG1k	8.08

a Calculated from antibody sequence with ProtParam by Expasy (Swiss Institute of Bioinformatics).^57^

### Agitation Studies

Antibody solutions were filtered using 0.2 μm Whatman Puradisc 13 PVDF filters (cytiva Marlborough, USA) into prerinsed and dried 2R glass vials (Schott, Mainz, Germany). The fill volume in the 2R vials was 1 mL in a total vial volume of 4.1 mL, unless otherwise stated. All vials were sealed with West FluoroTec stoppers (West Pharmaceutical Services, Exton, USA), crimped, and mounted vertically on a digital orbital shaker (19 mm orbit; Heathrow Scientific, Illinois, USA). Agitation was performed at room temperature with 300 rpm for 6 h. Each sample was produced in triplicate by performing three agitation experiments each alongside three unstressed controls. A matched buffer control was subjected to identical handling. After agitation, all samples were stored at 4 °C and analyzed at room temperature within 36 h.

### Visual Inspection

Visual inspection was performed against nonglare black and white backgrounds under gentle inversion for at least 5 s. Samples were ranked according to the number of particles observed. Samples practically free of particles were differentiated from samples with fewer or more than 20 particles below ∼0.5 mm. Samples that showed particles bigger than ∼0.5 mm or turbid samples were classified as strongly aggregated (Table S1).

### Flow Imaging Microscopy

Flow-Imaging microscopy (FIM) was performed with a FlowCam 8100 (Yokogawa Fluid Imaging Technologies, Scarborough, USA) equipped with an 80 × 700 μm flow cell and a 10× objective. The flow rate was set at 0.15 mL/min with an auto image frame rate of 27 frames/s. Particle detection thresholds were set at 13/10 for dark/light pixels with a 3 μm separation from neighboring particles. Triplicates of 200 μL each were injected. Air bubbles and silicone oil larger than 8 μm were manually excluded from the analysis. Reported particle counts refer to those with equivalent spherical diameters (ESD) > 2 μm determined via VisualSpreadsheet 6 (version 6.0.4.300, Yokogawa Fluid Imaging Technologies, Scarborough, USA).

### FIM Data Clustering via Machine Learning

All images containing particles larger than 10 μm ESD obtained using FIM were used for clustering-based machine learning analysis.^58^ A ResNet-50 model,^59^ pretrained using the state-of-the-art self-supervised method SwAV^60^ on the ImageNet data set,^61^ served as the feature extractor for this analysis.

To ensure an unbiased evaluation, a data set of 1850 images per mAb was assembled, from which features were extracted using the ResNet-50 model to construct the feature database. Additional 250 images per mAb were reserved for evaluation. For each evaluation image, features were similarly extracted, and cosine distances were calculated against all entries in the feature database to identify the five most similar images.^62^

### UV Spectroscopy

UV absorbance measurements at 280 nm for mAb concentration determination were performed in triplicate on a Nanodrop 2000c (Thermo Fisher Scientific, Waltham, USA). The concentration was calculated using the extinction coefficients calculated with ProtParam, Expasy (Swiss Institute for Bioinformatics, Lausanne, Switzerland).^57^

### Size-Exclusion Chromatography

Size-exclusion chromatography (SEC) was carried out with a Superdex 200 Increase 10/300 GL (cytiva Marlborough, USA) column on a Waters Arc HPLC system equipped with an Arc Premier 2489 UV/vis detector (Wyatt | Waters, Santa Barbara, USA). The elution of unstressed mAb was additionally measured with an Optilab rEX refractive index (RI) and a miniDawn Treos multiangle light scattering (MALS) detector (Wyatt | Waters, Santa Barbara, USA) for molecular weight confirmation. All runs were performed in duplicate at 1 mL/min flow for 30 min with 1× phosphate-buffered saline (PBS) (pH 7.4). 30 μg of protein were injected per run, and the UV signal was integrated via Astra Software (version 8.2.2.119 Wyatt | Waters, Santa Barbara, USA) to quantify the monomer and small soluble aggregate content.

### Differential Scanning Fluorimetry

Apparent melting temperatures (*T*_m_) of mAbs were determined via differential scanning fluorimetry (DSF). Triplicate 10 μL aliquots were filled into black 384-well PCR plates (HSP3866, Bio-Rad, Hercules, USA) and sealed with qPCR adhesive films (4ti-0560, Azenta, Burlington, USA). Thermal unfolding was monitored using a SUPR-DSF device (Protein Stable, Leatherhead, UK) with a linear heat ramp of 1 °C/min. Samples were excited at 280 nm, and emission spectra were collected from 310 to 420 nm. The barycentric mean (BCM) of the emission spectra was used to construct thermal unfolding curves, from which *T*_m_ was obtained as the maximum of the first derivative within the SUPR Suite software (version 4.8.0.0, Protein Stable, Leatherhead, UK).

### Modulated Scanning Fluorimetry

Modulated scanning fluorimetry (MSF) was conducted using the same experimental setup as for DSF (see above). Instead of a linear heat ramp, incremental heating and cooling cycles were operated and analyzed as previously described.^43^ Cycles consisted of a 5 min holding phase at 25 °C before heating to the target temperature with 10 °C/min. The target temperature was increased by 1 °C per cycle starting from 25 °C up to 105 °C. Target temperature was held for 1 min before cooling down to 25 °C again. Emission spectra were repeatedly collected at 25 °C and at target temperatures, allowing the construction of unfolding and nonreversibility curves based on the BCM. Origin Pro 2024 (Northampton, USA) was used to determine *T*_nr_ from the nonreversibility curve, defined as the 10% offset from the baseline. Offset from the baseline was determined manually for measurements with a baseline noise or shift >10%.

### Dynamic Light Scattering

Aggregation onset temperatures (*T*_agg_) were determined using a DynaPro DLS plate reader (Wyatt | Waters, Santa Barbara, USA). Triplicate 30 μL mAb samples were loaded into 384-well LoBase plates (Aurora Microplates Inc., Carlsbad, USA) and sealed with silicone oil. Measurements were performed during a linear heat ramp (25–70 °C, 0.1 °C/min) with three DLS acquisitions of 5 s each. The increase of the apparent hydrodynamic radius (*R*_h_) with temperature was fitted for the onset within the Dynamics software (version 8.4.1.460, Wyatt | Waters Santa Barbara, USA) to obtain *T*_agg_.

The same DynaPro DLS plate reader (Wyatt | Waters Santa Barbara, USA) was used to determine the diffusion self-interaction parameter *k*_D_. Seven dilutions of each mAb between 1 and 10 mg/mL were filled in 384-well LoBase plates (Aurora Microplates Inc., Carlsbad, USA) and sealed with silicone oil. Twenty DLS acquisitions of 5 s were performed for three replicates of each dilution at 25 °C. *k*_D_ was calculated using Dynamics software (version 8.4.1.460, Wyatt | Waters Santa Barbara, USA) as the slope of the protein concentration dependence of the diffusion coefficient D according to

$$D=D_{0}(1+k_{D}\times c)$$

where *D*_0_ is the diffusion coefficient at infinite dilution and *c* the protein concentration.

### Hydrophobic Interaction Chromatography

Hydrophobic Interaction Chromatography (HIC) was performed on a Dionex Summit 2 system equipped with a UVD170U detector (Dionex, Sunnyvale, USA). Separation was achieved on a Sepax Proteomix HIC butyl-NP5 column (Sepax Technologies, Delaware, USA) equilibrated at 30 °C. A linear 30 min gradient was applied (from 100% mobile phase A to 100% B), followed by a 10 min isocratic step with mobile phase B (flow rate 0.5 mL/min). Mobile phase A consisted of 1.8 M ammonium sulfate in 0.1 M sodium phosphate buffer (pH 6.5). Phase B was 0.1 M sodium phosphate buffer (pH 6.5). Before injection, the antibody samples were diluted to 0.22 mg/mL with phase A and 60 μL were injected. Each sample was analyzed twice. Retention times were determined using Chromeleon software (Thermo Fisher Scientific, Waltham, USA).

### ReFOLD Assay

The aggregation propensity during isothermal unfolding and refolding from a denaturant was assessed using the ReFOLD assay. Triplicate antibody samples (0.45 mg/mL, 100 μL each) were dialyzed against 1.8 mL of 8 M urea dissolved in 10 mM acetate buffer, pH 5, with 0.9% NaCl using Pierce 3.5 kDa MWCO microdialysis devices (Thermo Scientific, Waltham, USA). Dialysis was performed at room temperature with buffer exchanges after 4 and 8 h. After 24 h of dialysis against the urea-containing buffer, samples were dialyzed likewise against 1.8 mL urea-free 10 mM acetate buffer, pH 5, with 0.9% NaCl. Finally, the samples were recovered from the microdialysis devices, weighted on a microbalance, and sample weight was adjusted to 200 mg using 10 mM acetate buffer, pH 5, with 0.9% NaCl. Subsequently, the samples were analyzed by SEC using a Superdex 200 increase 10/300 GL column (cytiva Marlborough, USA) and a Dionex Summit 2 system equipped with a UVD170U detector (Dionex, Sunnyvale, USA) and Chromeleon software (Thermo Fisher Scientific, Waltham, USA). Duplicates (60 μL) were injected and eluted with 1X PBS (pH 7.4) at 1 mL/min over 40 min. The relative monomer yield (RMY) was calculated by dividing the monomer peak area of the refolded antibody by the monomer peak area of the antibody reference that was not unfolded and refolded but diluted accordingly.

### Optical Observations of Thin Liquid Films in the Capillary Cell

A capillary cell was used to observe the behavior of thin foam films.^63^ The films were formed in a capillary with radius of 1.25 mm by drawing in a mAbs solution, which was obtained by thawing the sample in a water bath at a temperature of 25–27 °C for 30 min, through a side orifice. The films were observed in reflected light with an optical microscope Leica DM RXE (Leica Microsystems GmbH, Wetzlar, Germany) equipped with a long-distance objective N PLAN 20x/0.4 (Leica Microsystems GmbH, Wetzlar, Germany) and a 5.1 M Video Biological Microscope Digital Camera 55FPS LCMOS (ToupTek Europe, UK). The typical radius of the foam films formed in this capillary cell was ≈0.125 mm. The film thinning behavior and the stability of the foam films were studied for 10 min after its formation in the closed cell. Subsequently, the cell was opened to the atmosphere, and the film stability was monitored for an additional 5 min. To assess the drainage dynamics and stability of the foam films, we also measured the time required for the appearance of the first black spot in the film – an indicator of local thinning. Shorter black spot formation times correspond to faster film drainage and reduced structural resistance. Such film behavior is typically associated with more mobile interfacial layers. At least three independent films were observed for each mAb solution.

### Foam Formation and Optical Observations of Thin Liquid Films and Bubbles in Real Foams

After thawing in a water bath at a temperature of 25–27 °C for 30 min, 0.2 mL of each sample were pipetted into a 1.5 mL centrifuge tube and manually shaken 20 times to generate foam and to assess the influence of shaking on the surface properties of the mAbs. The resulting foams were evaluated based on their stability and volume, with a scoring system applied to each foam. Optical observations of the bubbles and foam films in the obtained foams were performed in reflected or transmitted light, using a microscope Axioplan (Zeiss, Oberkochen, Germany), equipped with long-distance objectives: Plan-Neofluar 10x/0.3; Zeiss Epiplan 20x/0.40; Zeiss Epiplan 50x/0.50. Depending on the film thickness, the light reflected by the film has different colors. From the color and the intensity of the light reflected from the foam film, one can assess the film thickness. The films that are thicker than 100 nm appear colored. The films with a thickness of about 100 nm appear white (bright), those with a thickness of about 50 nm appear gray, and those whose thickness is under 30 nm appear dark. With the help of these observations, we were able to determine the location of the protein aggregates in the foams - whether they were adsorbed at the surface of the foam films or remained in the solution, freely floating around the bubbles. All experiments were conducted at ambient temperature (25 °C).

### Surface Tension and Interfacial Rheology by Drop Shape Analysis

Samples were first thawed in a water bath at 25–27 °C for 30 min to ensure complete melting and homogenization. Then, a metal capillary (volume: 150–300 μL) was filled with the solution, and surface rheological properties were determined using the oscillating drop method module integrated into the DSA 100 automated instrument (Krüss GmbH, Hamburg Germany).^64^ The surface tension was measured using the Drop Shape Analysis (DSA). Surface tension was converted into surface pressure (π) by subtracting the surface tension after mAb adsorption from the surface tension of the bare air–water interface. Subsequently, surface area oscillations were applied to the drop with a deformation amplitude of 1–2% and a period of 5 s. The measurement of surface tension was then continued without oscillation up to ≈900 s. At this point, the drop was once again subjected to oscillations using the same parameters (1–2% deformation for 5 s) to evaluate the surface dilatational modulus.

## Results

### Antibodies Exhibit Markedly Different Aggregation Propensity during Agitation

Our initial objective was to assess whether mAbs exhibit significant intrinsic differences in aggregation propensity when stressed by agitation. To generate a robust benchmark data set, we conducted controlled agitation experiments on ten mAbs reformulated in a surfactant-free acetate buffer with 0.9% NaCl, mimicking common infusion bag conditions. We then assayed the stressed samples with several orthogonal techniques that cover a broad aggregate size range.

Flow imaging microscopy (FIM) revealed that agitated mAbs contain very different concentrations of micrometre-sized aggregates (ca. 2–100 μm) with characteristic morphologies (Figure 1a). BEVA and NIVO formed the highest number of particles upon agitation, exceeding other mAbs by more than 10-fold. In contrast, VEDO, TRAS, and ISAT showed no increase in micrometre-sized particles. Particle formation trends were consistent across different micrometre-size ranges (Figure S1). In addition, some visible aggregates (ca. >100 μm)^65^ were observed in all samples (Table S1), and BEVA and NIVO turned turbid upon agitation (Table S1).

**Figure 1:**
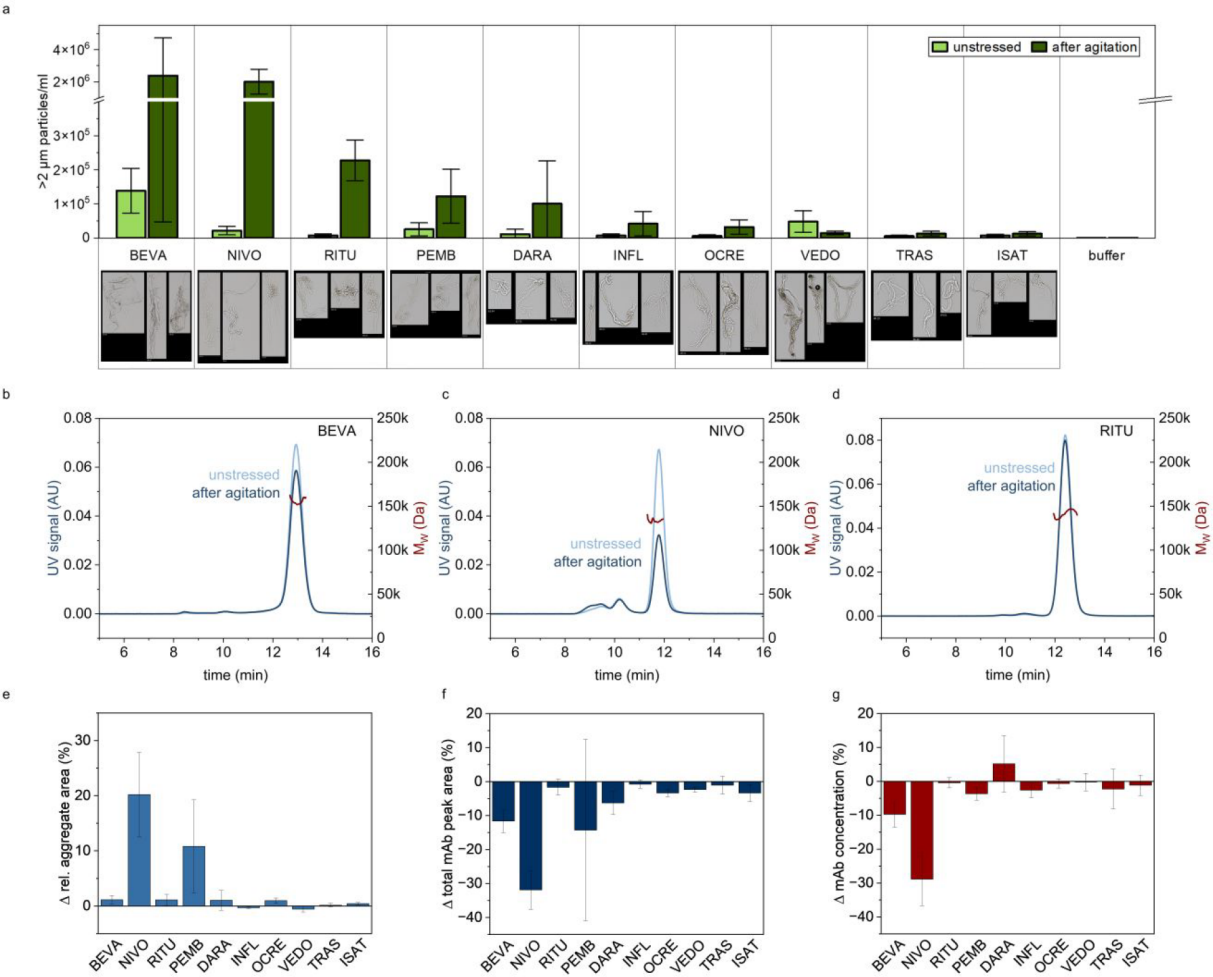
Different degrees of mAb aggregation induced by agitation stress. (a) Micrometre-sized aggregate (ca. 2–100 μm) concentration determined with FIM before and after agitation. Mean of nine values (triplicates, each replicate was measured three times) with SD. Below exemplary images of mAb aggregates detected after agitation. (b–d) Exemplary chromatograms for BEVA, NIVO, and RITU acquired with SEC-MALS before and after agitation. (e) Change in relative SEC-UV aggregate peak area upon agitation. Mean of six values (triplicates, each replicate was measured twice) with SD. (f) Change of total SEC-UV peak area (%) upon agitation. Mean of six values (triplicates, each replicate was measured twice) with SD. (g) Relative mAb concentration changes upon agitation determined with UV spectroscopy. Mean of nine values (triplicates, each replicate was measured three times) with SD.

UV spectroscopy showed that the concentration of BEVA and NIVO was reduced after agitation (Figure 1g), consistent with the substantial aggregate formation detected by FIM. Furthermore, we assayed the relative aggregate content and total mAb recovery with size-exclusion chromatography (SEC) (Figure 1e,f). Exemplary chromatograms can be found in Figures 1b–d and S2. In agreement with other methods, BEVA and NIVO showed a substantial decrease in total mAb recovery (Figure 1f). Interestingly, the relative signal coming from small soluble aggregates increased only for NIVO and PEMB and did not change for the other mAbs (Figure 1e). This indicates that agitation likely leads to bigger aggregates that are beyond the size range of the SEC column.

Overall, the ten model mAbs exhibit significant differences in their agitation-induced aggregation and set the benchmark for testing stability predictive methods.

### Standard Developability Parameters Fail to Predict Agitation-Induced Antibody Aggregation

We asked if a standard developability assessment focused on aggregation propensity prediction can identify mAbs sensitive to agitation. Therefore, we characterized the ten antibodies with a battery of established developability assays that provide complementary information.

DSF was used to probe the apparent melting temperature (*T*_m_), which is a proxy for the conformational stability (Figure 2a). For most mAbs, multiple transitions stemming from the different antibody domains could be resolved. The first melting temperature is around 65 °C, which can be attributed to the C_H_2 domain or an overlapping unfolding of a less stable Fab together with C_H_2.^66^ In seven out of the ten mAbs, we could observe a second melting temperature that indicates a Fab that unfolds at a higher temperature compared to C_H_2 (Figure 2a). In addition, we used MSF to investigate the refoldability upon structural perturbations caused by different temperatures (Figure 2b). RITU shows the highest nonreversibility temperature (66.3 °C), while the value is lowest for PEMB (57.1 °C). The aggregation onset temperatures (*T*_agg_) from DLS reveal a slightly different stability ranking, where INFL has the lowest *T*_agg_ (50.7 °C) and TRAS the highest *T*_agg_ (69.6 °C) (Figure 2c). Noteworthily, most *T*_nr_ and *T*_agg_ values are in the range between 60 and 65 °C, indicating that the nonreversible changes are likely because of aggregation.^47^

**Figure 2:**
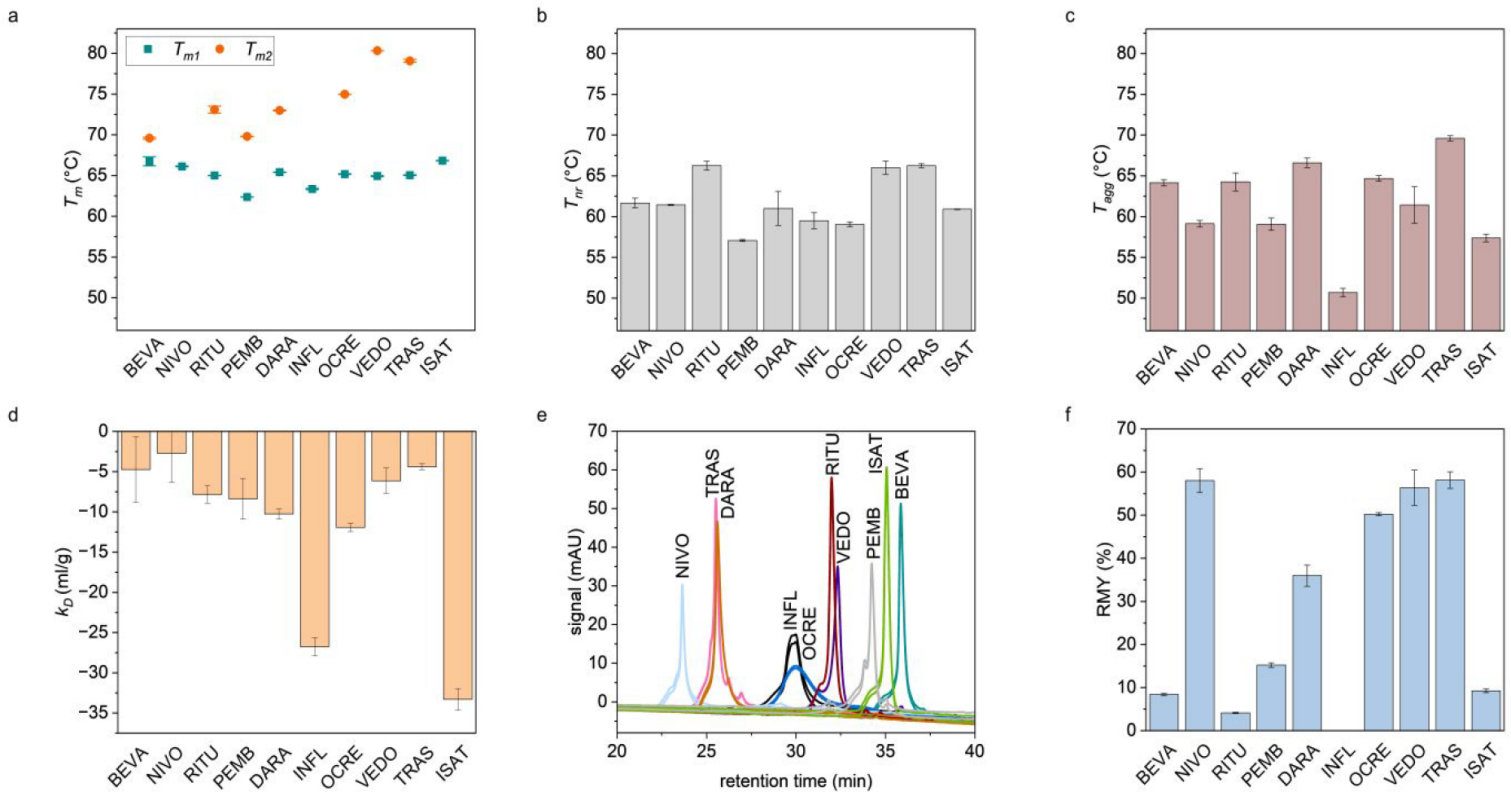
Standard developability parameters of the model IgGs. (a) *T*_m_ from DSF. Mean of triplicates with SD. (b) *T*_nr_ from MSF. Mean of triplicates with SD. (c) *T*_agg_ from DLS. Mean of triplicates with SD. (d) *k*_D_ from DLS determined from triplicates with SD. (e) Overlay of chromatograms acquired from HIC (duplicates). (f) Relative monomer recovery from the ReFOLD assay. Mean values of six measurements (each triplicate was measured twice).

In addition to the thermal-ramp methods, we used several isothermal orthogonal techniques. To probe the colloidal stability, we determined the *k*_D_ from DLS. All mAbs show negative *k*_D_ values, indicating attractive behavior in the acetate buffer with 0.9% NaCl (Figure 2d). The *k*_D_ values are negative likely due to charge screening by NaCl.^56,67^ INFL and ISAT show the lowest *k*_D_ values in saline. Furthermore, we used HIC to probe the hydrophobicity. NIVO and BEVA show the lowest and highest hydrophobicity according to their retention times (RT) (Figure 2e). Finally, to measure the non-native aggregation propensity during refolding from denaturants, we used the ReFOLD assay. The highest monomer recovery was obtained for NIVO, VEDO, TRAS, and OCRE, indicating that these mAbs exhibit the lowest aggregation during refolding from urea (Figure 2f).

Next, we wondered whether there are correlations between the biophysical descriptors and the aggregation of the mAbs caused by agitation. Despite the apparent differences between the complementary descriptors, none of the parameters correlated with the number of agitation-induced micrometre-sized aggregates, antibody concentration change, or formation of small soluble aggregates (Figure 3).

**Figure 3:**
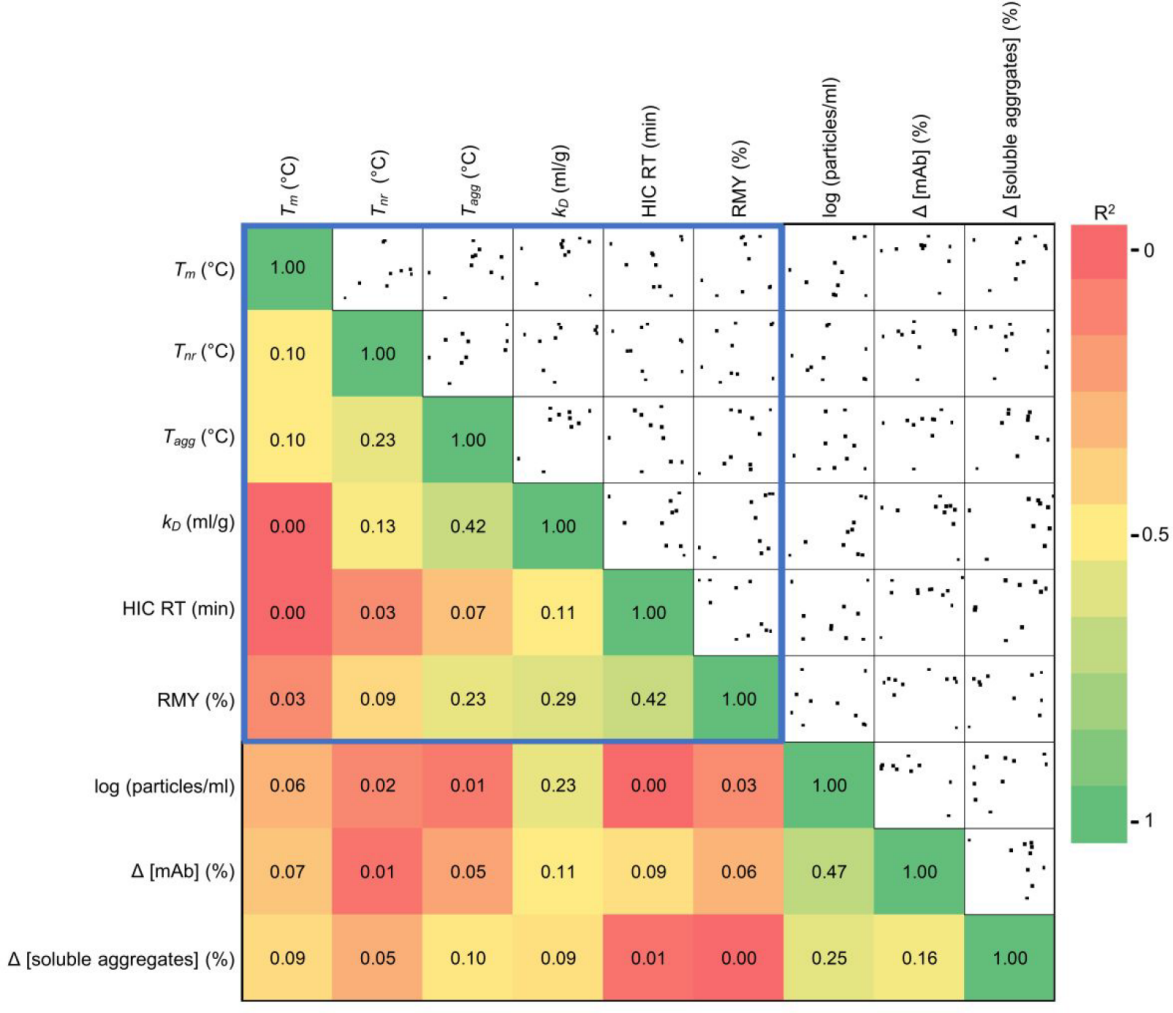
Lack of correlation between standard developability parameters (blue box) and aggregation upon agitation. The figure displays the coefficient of determination *R* ^2^ for linear correlation between each parameter. The distribution of data points is shown in the right part of the figure.

Therefore, although these basic biophysical parameters are valuable during developability analysis^32,33,35,50,51^ they do not provide predictive information on mAb stability during agitation.

### Agitation Causes Antibody Aggregation Primarily at the Air–Liquid Interface

After observing that standard developability parameters do not correlate with agitation stability, we sought to investigate the mechanism of agitation-induced antibody aggregation further.

We first studied thin liquid films, which form when the surfaces of two air bubbles are in proximity to each other. These films provide direct information about the presence of aggregates on the surface and have been widely studied in the context of foam stability.^68–71^ We observed multiple aggregates in the thin liquid films for 9 out of 10 mAbs, as illustrated for BEVA (Figure 4a,b) (images of thin liquid films of all mAbs studied are presented in Table S2). The only exception was TRAS – its thin liquid films were unstable and were quickly destroyed during thinning, preventing imaging.

**Figure 4:**
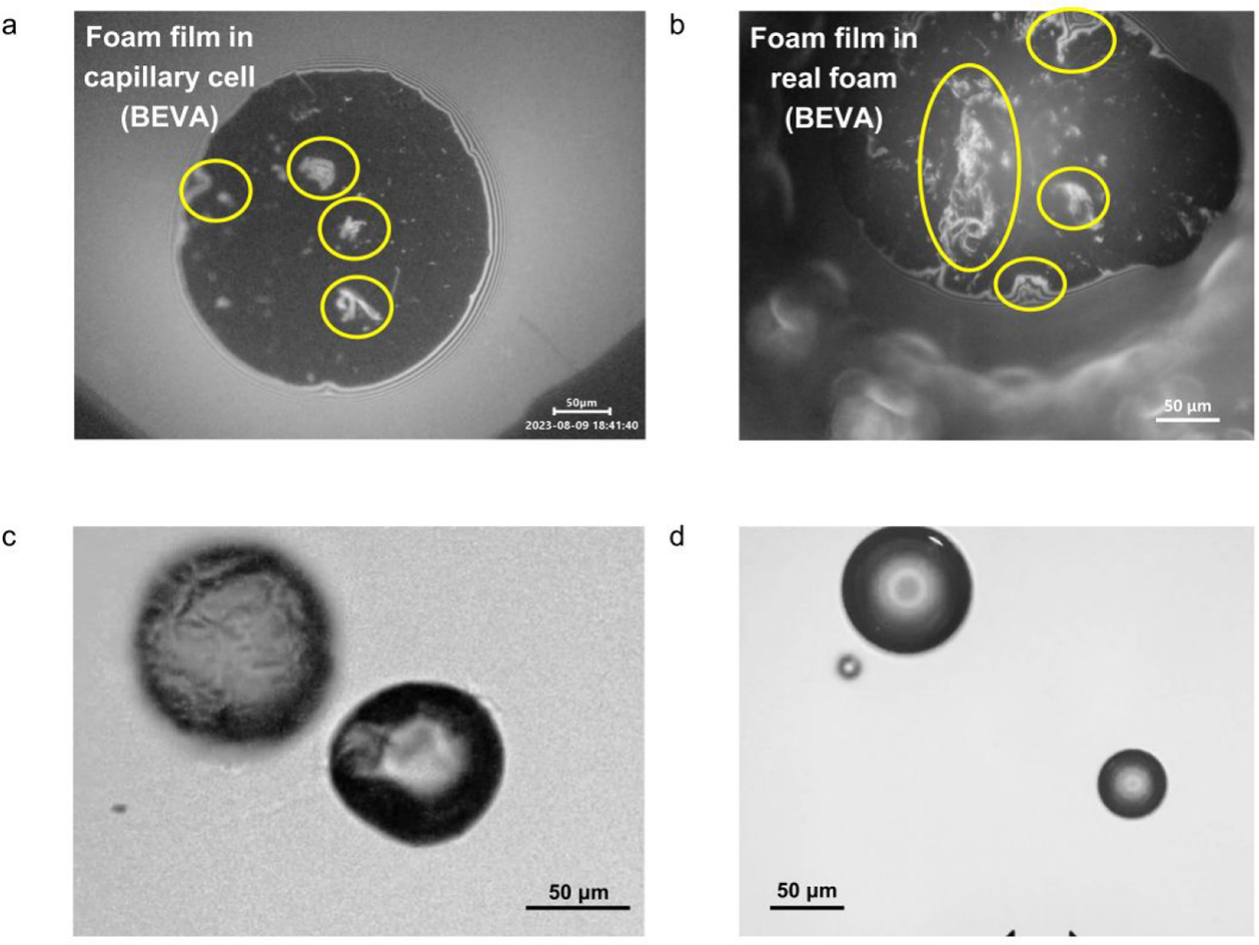
Interaction of mAb solution and air. (a,b) Foam films of BEVA formed in (a) capillary cell or (b) real foam. The black color of the film indicates thickness < 30 nm, whereas the white or colored spots show thicker areas, due to aggregate adsorption or entrapment. Aggregates are encircled in yellow. (c,d) Images of bubbles in agitated solutions of (c) BEVA and (d) TRAS.

As aggregates can be either adsorbed or trapped in the films, we also performed optical observations of agitated mAbs solutions which contained air bubbles. The results showed considerable adsorption on the bubble surface of some of the mAbs, causing wrinkling and bubbles with nonspherical shape, as shown for BEVA (Figure 4c). Similar behavior was found for INFL, OCRE and VEDO. In the case of the other six mAbs studied, TRAS, ISAT, DARA, NIVO, PEMB and RITU, the air bubbles remained spherical, suggesting less pronounced mAb adsorption (a representative image of TRAS is presented in Figure 4d). Images of all mAbs studies are presented in the supplementary (Table S3). The measured black spot formation times (Table S5) did not correlate with mAb stability.

To further confirm the role of the air–liquid interface in agitation-induced aggregation, we performed additional experiments where BEVA and NIVO were agitated in completely filled vials without a headspace, ensuring that no air–liquid interface was present. The results showed more than 10-fold reduction in the aggregate count determined with FIM when the air–liquid interface was eliminated (Figure S3a). Congruently, soluble mAb concentration (Figure S3f) and monomer content (Figure S3b,c,e) were retained as determined with UV spectroscopy and SEC. Small soluble aggregates were not formed during agitation in the absence of air, in contrast to aggregate formation upon agitation with an air headspace (Figure S3d). In addition, machine learning analysis of the FIM data from agitation of ten mAbs in the presence of air showed that the aggregate morphology of all mAbs is highly similar (Table S4). Classification experiments using a majority vote among the five most similar mAbs yielded an accuracy of only 26.2%. Accordingly, machine learning methods cannot reliably distinguish between aggregates from different mAbs, indicating highly similar aggregate appearances. This supports the assumption of a common aggregation mechanism via interfacial film formation and rupture.

### Surface Pressure Combined with Surface Elasticity Correlate with Antibody Aggregation

After confirming that the air–liquid interface triggers mAb aggregation during agitation, we set out to evaluate different surface characteristics as potential developability indicators. For example, surface tension and interfacial rheology have been proposed as predictive tools for interfacial stability based on a limited number of molecules.^19,72,73^ Surface pressure (π) measurements at 600 s showed that mAbs can be roughly divided in two equally sized groups, one with low π (6.5–10 mN/m; BEVA, INFL, OCRE, VEDO, TRAS – marked with °) and one with approximately 2-fold higher π (16–19 mN/m; NIVO, RITU, PEMB, DARA, ISAT) (Figure 5a and Table S5). Interestingly, 4 out of 5 of the mAbs in the low π group (all but BEVA) were relatively stable against agitation-induced aggregation (Figure 1a). Furthermore, 4 out of 5 mAbs (all but ISAT) of the high π group exhibit more aggregation due to agitation (Figure 1a). Hence, the results obtained suggested a qualitative correlation between π and interfacial stability, with one outlier in each group.

**Figure 5:**
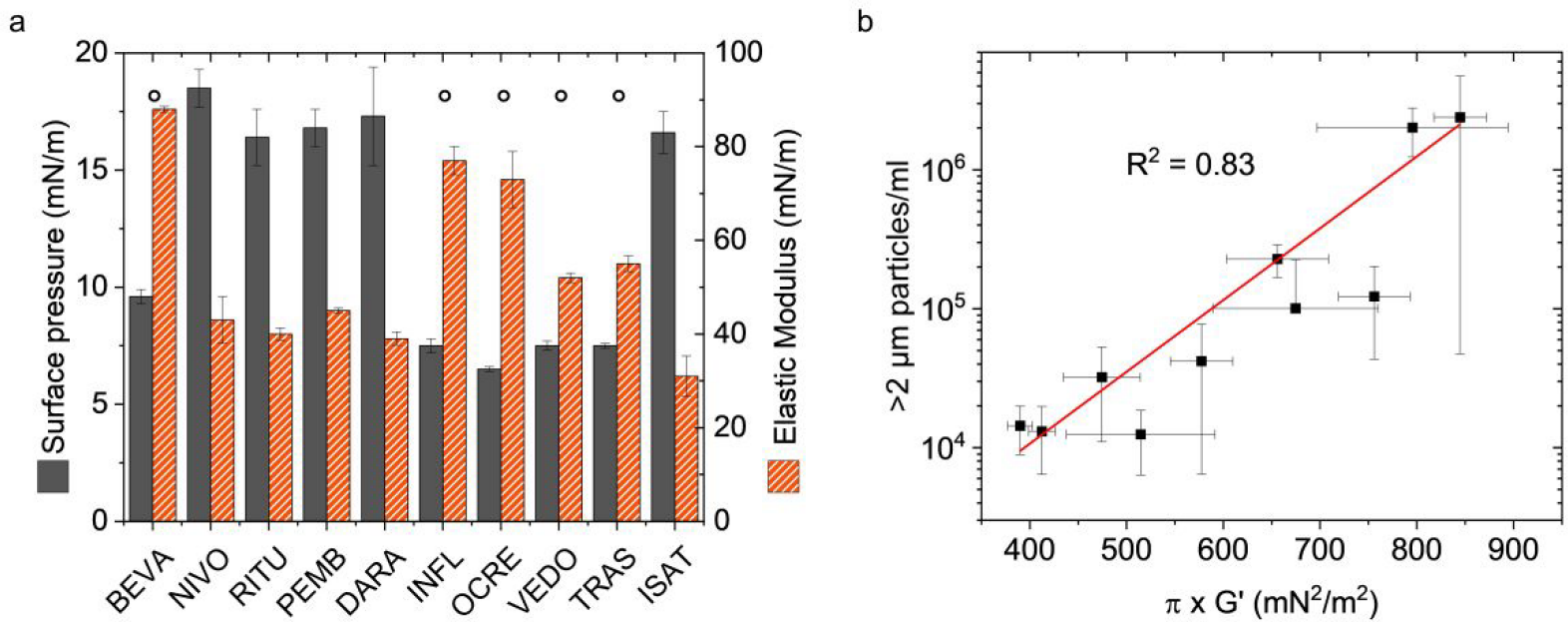
Surface properties of mAb solutions. (a) Surface pressure and elastic modulus of the studied mAbs measured after 600/900 s. ° marks the group of mAbs with low π and high *G*′. (b) Correlation between aggregate concentration after agitation and the product of surface pressure and elastic modulus of the studied mAbs.

Furthermore, the surface elastic modulus (*G*′) measured after ≈900 s showed a similar picture (Figure 5a and Table S5). The mAbs can be assigned to two groups (low and high *G*′), with low values generally correlating with low stability and high aggregate concentrations after stress (except for ISAT), whereas high values indicated better stability and low aggregate concentrations (except for BEVA). Therefore, we found that both π and *G*′ can be correlated to mAbs interfacial stability, but only on a semiqualitative level due to the outliers present in both cases (Figure S4). However, mAbs aggregation correlated reasonably well (*R* ^2^ = 0.83) with the combination of the two parameters (Figure 5b). High stability (less than 10^5^ aggregates/mL) was observed for mAbs with π × *G*′ < 600 mN^2^/m^2^, whereas higher π × *G*′ values clearly identified and ranked mAbs with lower stability to interfacial stress.

## Discussion

Interfacial stress caused by air–liquid interfaces is inevitable during the life cycle of an antibody drug. Our results illustrate that the air–liquid interface is the main driving force for mAb aggregation during agitation (Figure S3), and we show that intrinsic agitation stability varies significantly between mAbs. Unsupervised image clustering analysis confirmed that aggregates formed due to agitation have highly similar morphologies, in accordance with a common aggregation mechanism (Table S4). The clustering-based machine learning analysis showed that the employed model was able to group mAbs with similar structural properties, as the particles identified as similar within the feature set also appeared visually consistent. When applying a classification strategy based on a majority vote among the five most similar mAbs, the overall accuracy was only 26.2%. Accordingly, particles from different mAbs were frequently misclassified as one another, suggesting that the stressor type is the primary driver of aggregate morphology, rather than mAb-specific properties. Other studies have also found that the morphology of the aggregates is linked to the stress type.^74–82^

The behavior of mAbs at the air–water interface is essential for stability.^7,11,73,83–85^ Amphiphilic mAbs can adsorb to the interface and form viscoelastic films. Within this film, rearrangements and interprotein interactions can be energetically favored but can promote partial unfolding of the protein into β-sheets and the formation of aggregates that can be shed into the bulk solution upon shearing.^6–8,18–26^ Imaging of thin liquid films formed between air bubbles (Figure 4a,b) and of bubble dispersions in mAbs solutions (Figure 4c,d) confirmed that some of the protein aggregates remain adsorbed on the air–water surface, whereas others are ultimately shed as aggregates into the bulk solution under agitation.^7,9,21,86^

Antibody hydrophobicity, colloidal and conformational stability are expected to influence interfacial aggregation and particle shedding.^6,20,87^ We therefore tested whether biophysical parameters describing these properties can predict agitation-induced aggregation, which occurs mostly at air–liquid interfaces.^7–9,15,21,86^ We showed that none of the tested “bulk” descriptors correlates with the aggregates formed due to agitation (Figure 3). This aligns with earlier findings indicating the limited usability of developability parameters such as *k*_D_, *T*_m_ and hydrophobicity for stability prediction during agitation.^50,73,88,89^ Next to interfacial instability, electrostatic interaction, π-stacking, and hydrophobic interaction driven by surface patches of mAbs can also cause self-interaction and unspecific binding.^90–92^ Jain et al. have published data for 137 antibodies in clinical stages using eight different assays for determining self- and cross-interaction. Their data set includes results for nine of the ten mAbs that we tested here.^33^ We therefore investigated correlations of their results with the aggregation at the air–water interface. However, self-interaction and unspecific binding assays did not show predictive power for particle formation upon agitation (all *R* ^2^ < 0.34), potentially influenced by differences in the buffer system used and IgG isotype. In addition to those wet-lab techniques, in-silico tools are emerging for the determination of mAb developability. The Therapeutic Antibody Profiler is one of those commonly used tools. However, it is trained by commercial mAbs and limited to predictions at pH 7.4.^93^ Accordingly, the tool was not suited for the prediction of agitation-induced aggregation in our case (commercial mAbs in acetate buffer, pH 5 with 0.9% NaCl).

Because of the low predictive power of the standard developability assays, we examined mAb interfacial properties. Recent studies indicate that surface properties could directly correlate with protein stability. Mitropoulos et al. demonstrated that thermodynamically less stable proteins absorb more rapidly to the air–liquid interface, forming viscoelastic films with higher shear rheology.^87^ In line with this, Kannan et al. reported that a mAb with lower interfacial stability showed higher dilatational moduli than a more stable mAb.^19^ Extending these findings, the impact of NaCl as a clinically relevant diluent was investigated. An increase in particle formation at the interface in the presence of NaCl could again be linked to greater interfacial elasticity driven by greater interprotein interactions resulting from the screening of electrostatic interactions.^72^ In a broader study using 16 mAbs, Shieh and Patel found that the initial increase in π is a good predictor for mAb aggregation at the air–liquid interface.^73^ Pham et al. showed that both near-equilibrium π and elastic modulus correlate well with the aggregate concentrations generated during long-term storage stability.^94^

However, neither π, nor its increase in the first 10 s, or interfacial rheology (*G*′) correlated linearly with the agitation-induced aggregate formation in the ten mAb solutions in the current study while only semiqualitative relations could be found with π and *G*′ (Figures S4 and S5). Discrepancies could be related to the different surface characterization techniques used. For example, Shieh and Patel used a Wilhelmy plate tensiometer^73^ to measure surface pressure, compared to a Langmuir trough for Pham et al.^94^ and the drop shape analysis in the current study. We also have to note that it is not clear to what extent (if any) the mAb sets of the three studies overlap, as the mAb structures studied by Kannan et al., Pham et al. and Shieh and Patel were not disclosed.

As mentioned above, neither π nor interfacial elasticity alone was able to predict aggregation in the current study (Figure 5a). Also, foam volume (*R* ^2^ = 0.64) and the time for blackspot formation (*R* ^2^ = 0.39) did not seem suited as descriptive parameters (Figure S6 and Table S5). However, the combination of π near equilibrium (measurement after 900 s) and *G*′ was very powerful in discriminating well-behaved molecules from unstable candidates (Figure 5b). The surface pressure is indicative of the affinity of the proteins for the air–liquid interface, while the elastic modulus describes the strength of intermolecular interactions.^6,20,21,25,72,87,94^ We hypothesize that measures for both processes together are needed to describe interfacial stability.

The two parameters, π and *G*′, were measured via drop shape analysis – a method that allows the simultaneous determination of both characteristics in the same measurement with several repeats, while requiring relatively low sample volume (≈500 μL). However, the method requires specialized equipment and is relatively time-consuming.^95^ As throughput is currently low, the development of robust, high-throughput assays will be key to integrating interfacial stability analysis into early development.^52,96^

Our experiments were intentionally performed in a surfactant-free buffer to probe the intrinsic mAb stability during agitation. In practice, antibody adsorption and aggregation at the air–liquid interface are usually mitigated by surfactants. However, only three surfactants are used in approved antibody drugs – polysorbate 20 and 80, and poloxamer 188.^36^ These surfactants can act in various ways, such as competition with the antibody for surface adsorption or preferential binding to folded species. Interactions are complex, e.g., some depend on the critical micelle concentration, and some change with temperature. In some conditions, surfactants can even promote partial protein unfolding and aggregation.^37,38^ Kannan et al. showcased that clinically relevant NaCl addition promotes aggregation of a mAb in the presence of polysorbate 20 when compared with a low-ionic-strength buffer.^72^ Additionally, these nonionic surfactants are associated with major challenges such as impurities, degradation, and potential immune activation. Polysorbates are known to hydrolyze and autooxidise, losing their efficacy and causing harmful degradation products that can form (sub)­visible particles, act as aggregation cores, and promote protein oxidation. Poloxamer is more stable but less effective and also suffers from oxidation. In general, surfactants can promote the release of extractables and leachables such as di­(2-ethylhexyl) phthalate DEHP from PVC used in tubings and IV bags.^37–42,97^ Accordingly, it is desirable to select antibody drug candidates with high intrinsic stability against aggregation due to interfacial stress. Intrinsically stable mAb candidates could facilitate the formulation development and improve the safety during postproduction handling of mAbs in infusion bags.^9,11,16,32,33,72^

In terms of structure–stability relationships, most of the tested mAbs were IgG1, except for NIVO and PEMB, which are IgG4. Interestingly, both FIM and SEC indicated that NIVO and PEMB were among the more aggregation-prone mAbs during agitation. IgG4’s have already shown reduced thermal^98,99^ and colloidal stability in a head-to-head comparison with IgG1’s.^35,100^ Also, aggregate formation within the bulk solution can be promoted for the IgG4 isotype.^35,101,102^ Future work will have to elucidate the importance of the antibody isotype and the combination of different Fc and Fab fragments on the interfacial stability.

## Conclusions

In summary, we report that a standard developability analysis does not predict agitation-induced aggregation of mAbs driven by their inherent interfacial instability. In contrast, the combination of surface adsorption and interfacial rheology characteristics correlates well with the aggregation of mAbs caused by agitation in the presence of air–liquid interfaces. Specifically, we used the product of the surface elasticity (*G*′) with the difference between the surface tension of the bare air–water surface and the surface tension after mAb adsorption (π), to describe the important interplay between surface activity and interprotein interactions at the interface. Our findings emphasize limitations of established developability parameters and illustrate the need for dedicated developability assays that predict interfacial antibody stability.

## Supplementary Material



## Notes

Particle concentrations for ten mAbs before and after agitation, separated for different sizes, chromatograms from SEC-MALS acquired before and after agitation, results on mAb aggregation under agitation in the presence and absence of air, as well as correlations between aggregate concentrations and surface/foam properties (Figures S1–S6); results on the visual inspection of mAb solutions after agitation, pictures of foam films and air bubbles in mAb solutions, results from clustering of FIM images, as well as data on surface properties and blackspot formation for ten mAbs (Tables S1–S5) (PDF)

## Abbreviations

BCM: barycentric mean
*D*: diffusion coefficient
π: surface pressure
DLS: dynamic light scattering
DSA: drop shape analysis
DSF: differential scanning fluorimetry
ESD: equivalent spherical diameter
FIM: flow-imaging microscopy
*G*′: surface elastic modulus
HIC: hydrophobic interaction chromatography
IgG: immunoglobulin G
INN: international nonproprietary name
*k*_D_: diffusion self-interaction parameter
mAb: monoclonal antibody
MALS: multiangle light scattering
MSF: modulated scanning fluorimetry
PBS: phosphate-buffered saline
pI: isoelectric point
RT: retention time
*R*_h_: apparent hydrodynamic radius
RI: refractive index
RMY: relative monomer yield
SEC: size-exclusion chromatography
*T*_agg_: aggregation onset temperature
*T*_m_: apparent melting temperature
*T*_nr_: nonreversibility onset temperature
UV: ultraviolet
