# Supplementary material for: Predicting Agitation Stability of Monoclonal Antibodies during Developability Assessment
Source: Mol Pharm. 2026 Apr 24;23(6):3421–33. doi: 10.1021/acs.molpharmaceut.6c00092 (PMC13231419; doi:10.1021/acs.molpharmaceut.6c00092)
Supplement: Supplementary file 1 [file mp6c00092_si_001.pdf]

Supplementary Material

Predicting agitation stability of monoclonal antibodies during developability assessment

Michaela Cohrs[1], Nevena Pagureva[2], Utku Ozbulak[3.4], Wesley De Neve[3.4], Kevin Braeckmans[1], Stefaan De Smedt[1], Slavka Tcholakova[2], Zahari Vinarov[2] and Hristo L. Svilenov[5*]

[1] Laboratory of General Biochemistry and Physical Pharmacy, Ghent University, Ottergemsesteenweg 460, 9000 Ghent, Belgium

[2] Department of Chemical and Pharmaceutical Engineering, Faculty of Chemistry and Pharmacy, Sofia University, 1 J. Bourchier Ave., 1164 Sofia, Bulgaria

[3] Center for Biosystems and Biotech Data Science, Ghent University Global Campus, 119-5 Songdomunhwa-ro, Incheon, 21985, Republic of Korea

[4] Department of Electronics and Information Systems, Ghent University, Technologiepark-Zwijnaarde 126, 9052, Ghent, Belgium

[5] Biopharmaceutical Technology, TUM School of Life Sciences, Technical University of Munich, Emil-Erlenmeyer-Forum 5, 85354 Freising, Germany

* To whom correspondence should be addressed:

hristo.svilenov@tum.de https://orcid.org/0000-0001-5863-9569

phone: 0049 8161 71 2266

Figure S1

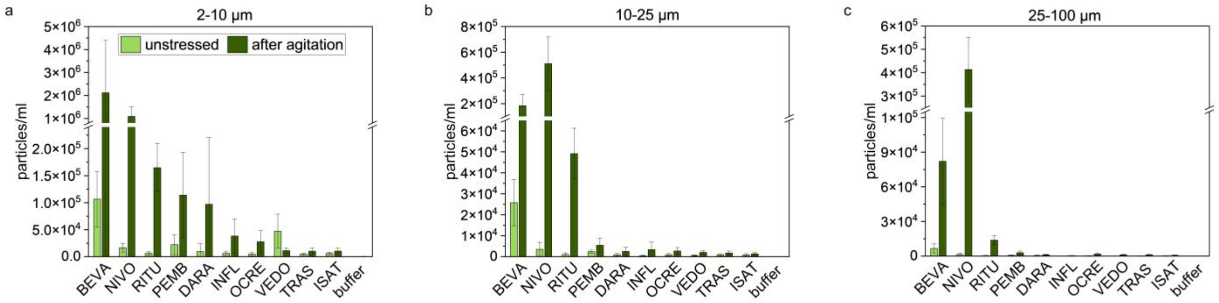

Figure S1. Particle concentrations quantified with FIM for ten mAbs before and after agitation. (**a**) 2-10 µm. (**b**) 10-25 µm. (**c**) 25-100 µm. Mean of nine values (triplicates, each replicate was measured three times) with SD.

Figure S2

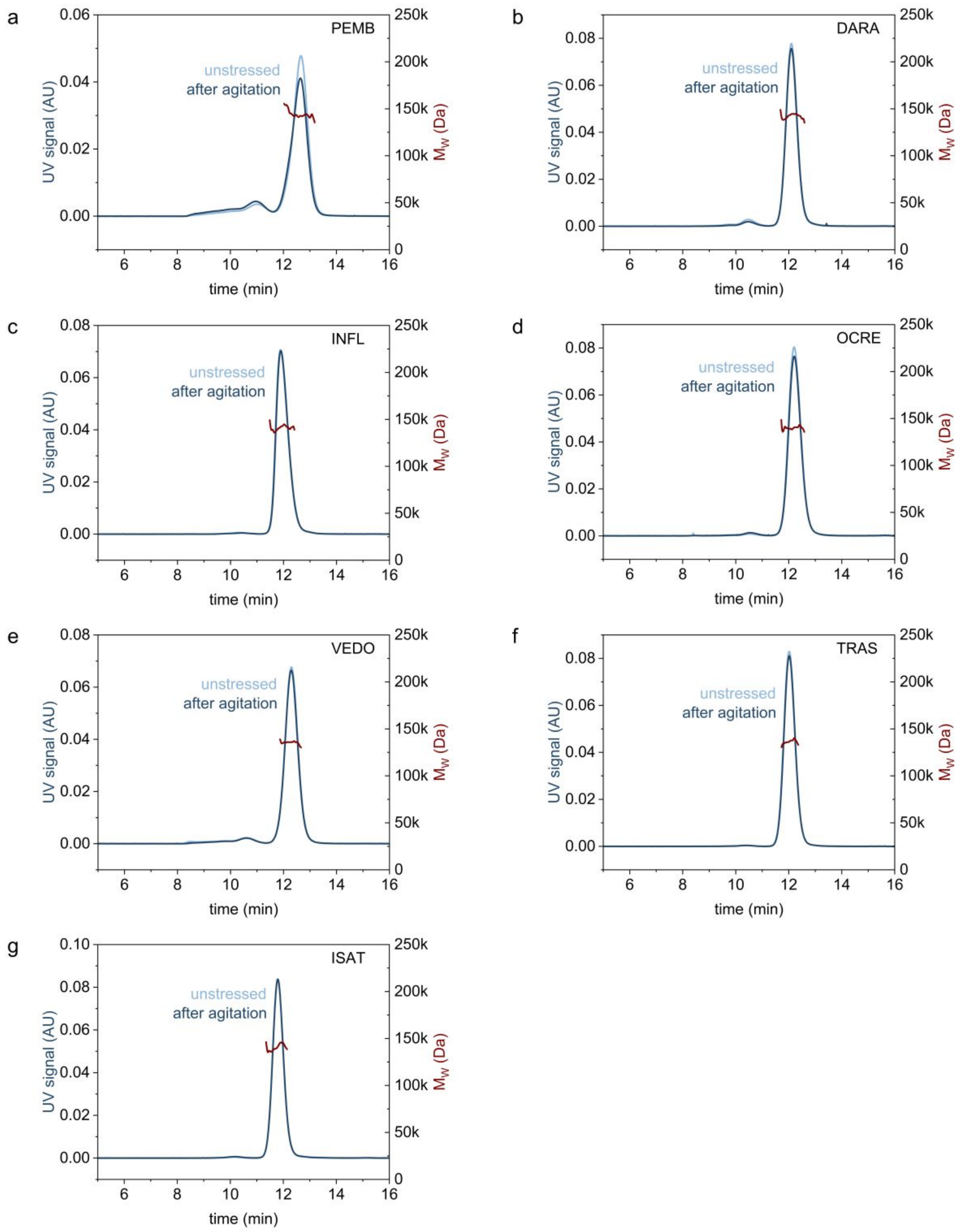

Figure S2. Exemplary chromatograms acquired with SEC-MALS before and after agitation.

Figure S3

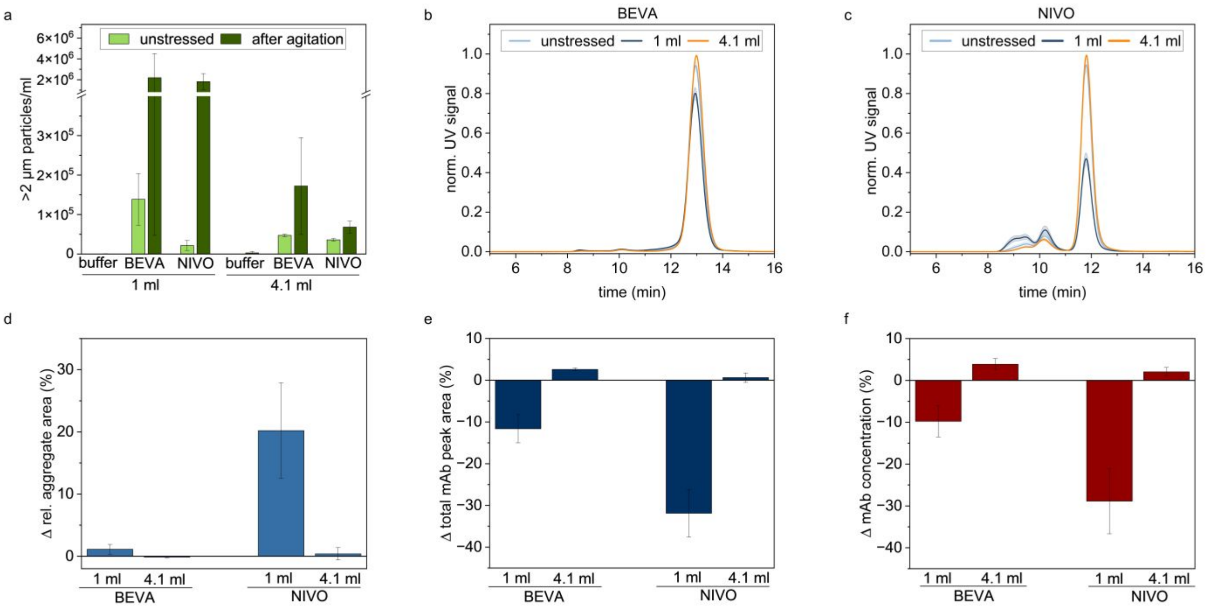

Figure S3. Change of mAb concentration and aggregates under agitation in the presence and absence of a headspace. (**a**) Micrometer-sized aggregates determined with FIM (ca. 2-100 µm) for BEVA and NIVO in the presence (1.0 ml) and absence (4.1 ml) of air-water interface before and after agitation (acetate buffer, pH 5, with 0.9% NaCl). Mean of nine measurements from triplicate are shown with SD. (**b**) Overlay of SEC chromatograms for BEVA before and after shaking of 1.0 ml and 4.1 ml. Mean of triplicates measured twice with SD. (**c**) Overlay of SEC chromatograms for NIVO before and after shaking of 1.0 ml and 4.1 ml. Mean of triplicates measured twice with SD. (**d**) Change in relative SEC-UV aggregate peak area upon agitation with 1.0 and 4.1 ml fill volume. Mean of triplicates measured twice with SD. (**e**) Change of total SEC-UV mAb peak area (%) upon agitation with 1.0 and 4.1 ml fill volume. Mean of triplicates measured twice with SD. (**f**) Relative concentration change (%) upon agitation via UV spectrophotometry. Mean of nine measurements from triplicates are shown with SD.

Figure S4

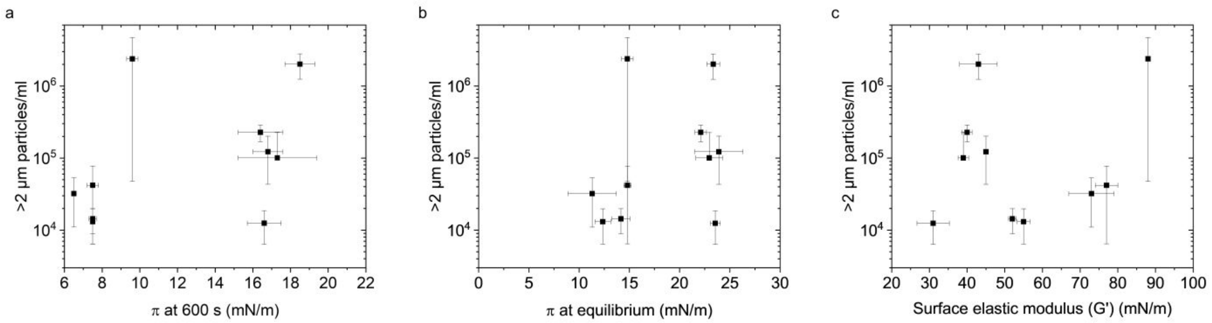

Figure S4. Correlations between aggregate concentration and surface properties. (**a**) surface pressure after 600 s (**b**) surface pressure at equilibrium (via extrapolation to infinity) and (**c**) surface elastic modulus, G' at 900 s.

Figure S5

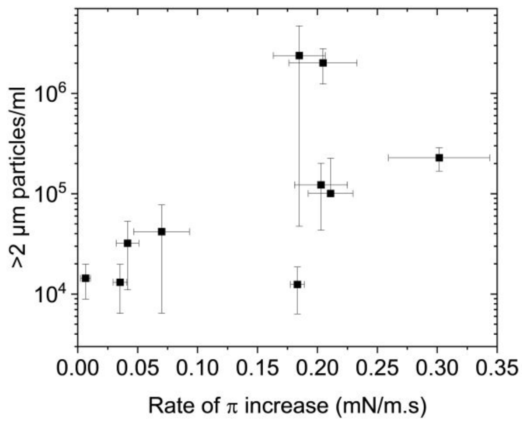

Figure S5. Lack of correlation between the rate of increase of surface pressure and aggregate concentration after mAbs agitation. The rate of increase of surface pressure was calculated from the slope of the first 10 seconds of the measurement (using the first five seconds and the equation in the paper of Shieh and Patel did not improve the correlation).

Figure S6

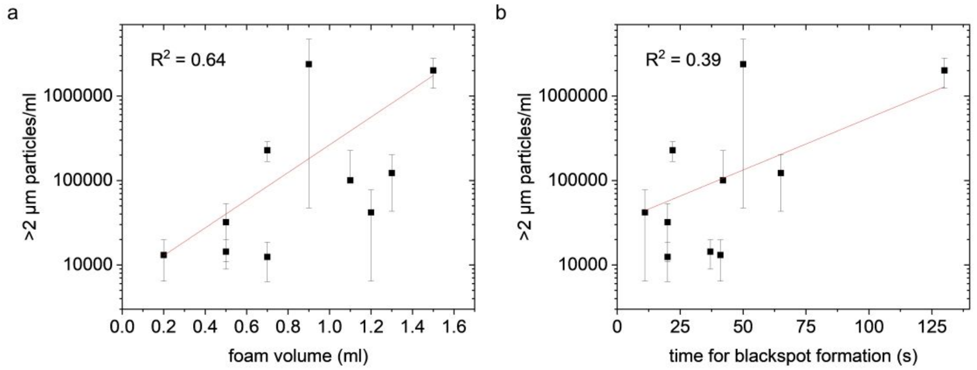

Figure S6. Lack of correlation between aggregate concentration and foam properties. (**a**) foam volume (**b**) time for blackspot formation.

Table S1

	unstressed			after agitation		
	replicate 1	replicate 2	replicate 3	replicate 1	replicate 2	replicate 3
1 ml fill volume						
buffer	0	0	0	0	0	0
BEVA	2	0	0	4	4	3
NIVO	2	0	0	4	4	4
RITU	1	0	0	2	2	3
PEMB	0	0	0	3	3	3
DARA	0	0	0	3	3	3
INFL	0	0	0	2	3	3
OCRE	0	0	0	3	3	3
VEDO	0	0	0	3	3	3
TRAS	0	0	0	2	3	3
ISAT	0	0	0	3	3	3
4.1 ml fill volume						
buffer	0	0	1	1	1	1
BEVA	2			3	1	3
NIVO	1			2	1	1

rating
0 - no particles detected
1 - <20 small particles (<<0.5 mm) detected
2 - >20 small particles (<<0.5 mm) detected
3 - particles > 0.5 mm
4 - turbid

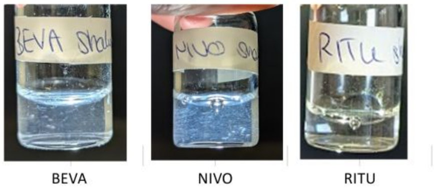

Table S1. Visual inspection for visible particle detection and turbidity. Examples of BEVA, NIVO, and RITU after agitation are shown on the lower right-hand side.

Table S2

	foam film immediately after formation	foam film after 10 minutes of observation	foam films in real foam
BEVA	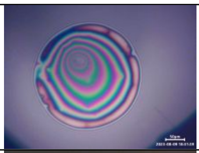	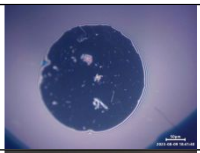	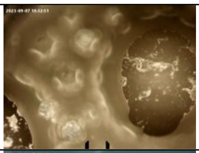
NIVO	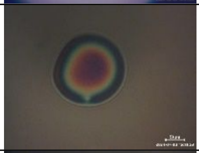	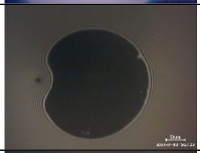	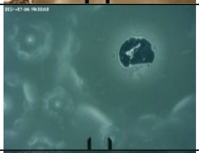
RITU	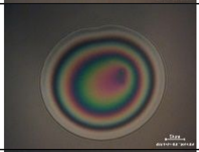	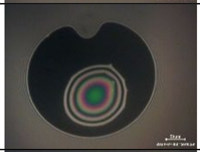	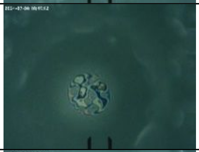
PEMB	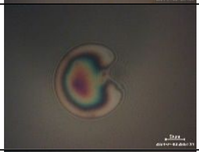	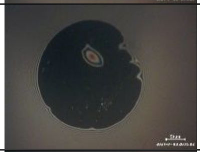	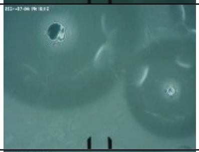
DARA	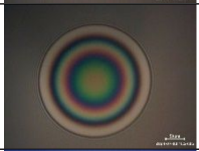	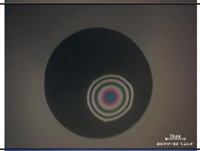	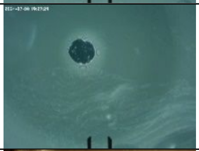
INFL	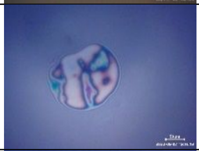	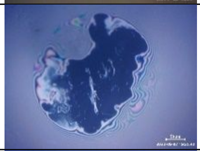	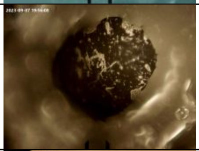
OCRE	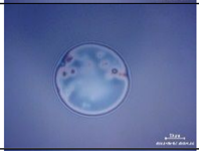	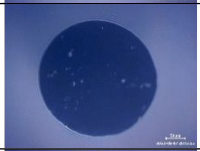	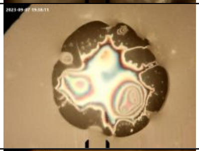
VEDO	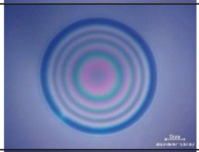	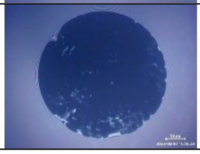	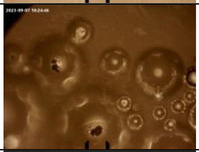
TRAS	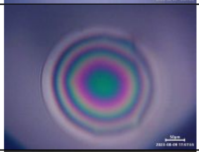	film rupture	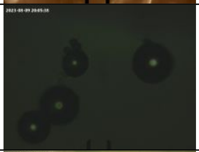
ISAT	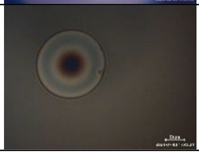	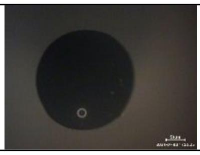	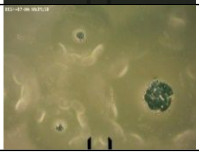

Table S2. Foam films pictures of mAb solutions. The first column presents the foam film immediately after formation in a Sheludko cell, the second column shows the same film after 10 minutes of observation and the third column presents images of foam films that were formed between bubbles in real foam.

Table S3

BEVA	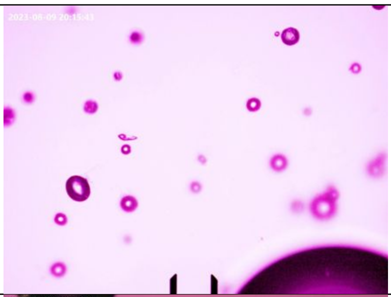	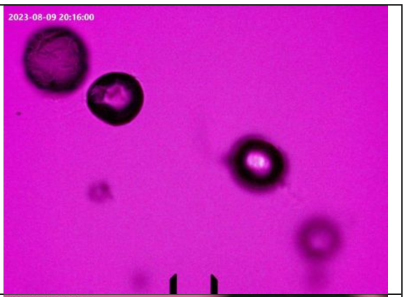
NIVO	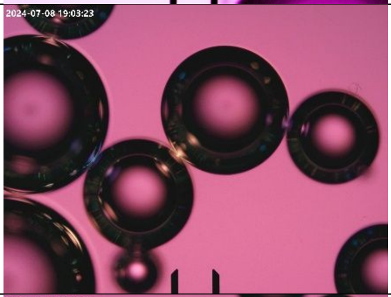	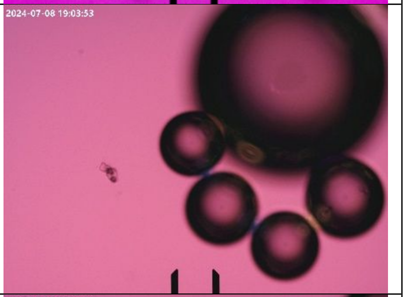
RITU	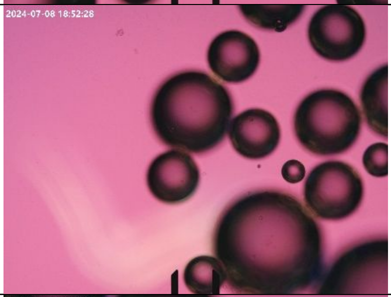	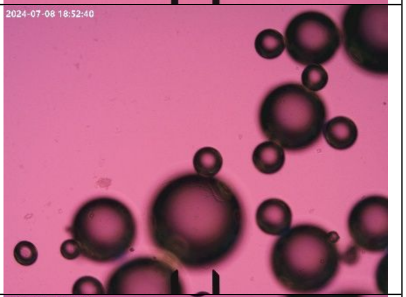
PEMB	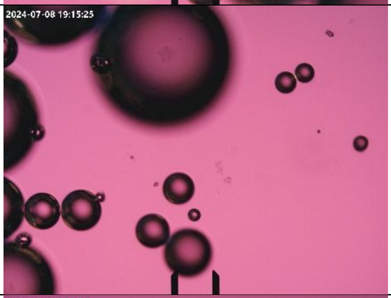	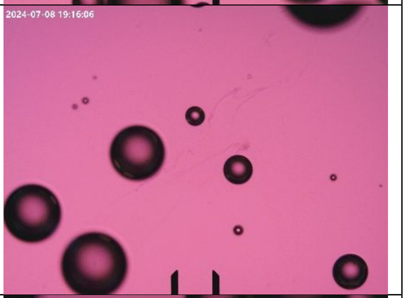
DARA	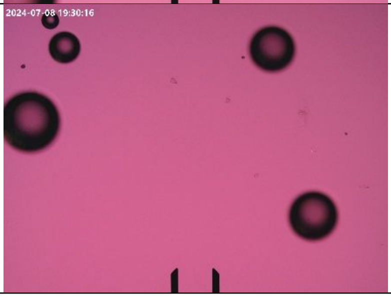	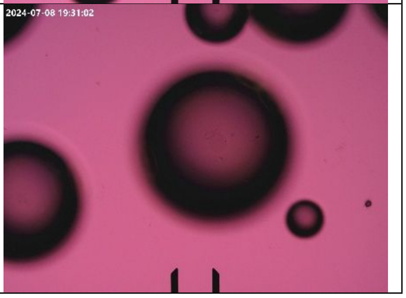

INFL	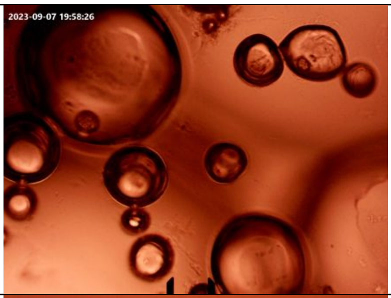	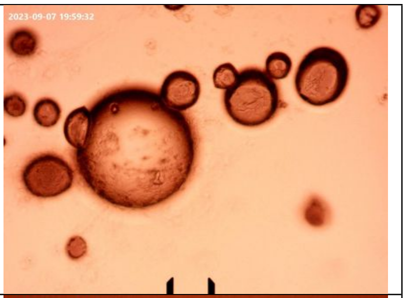
OCRE	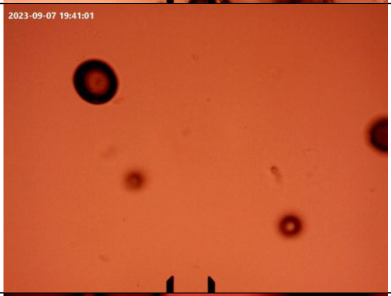	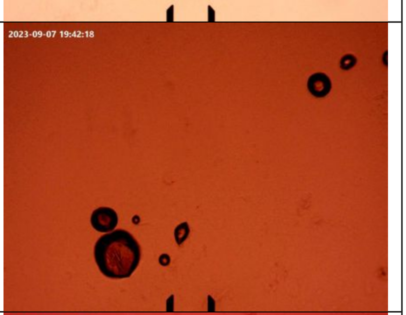
VEDO	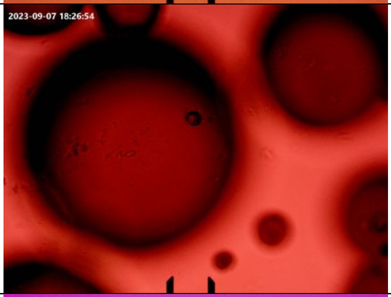	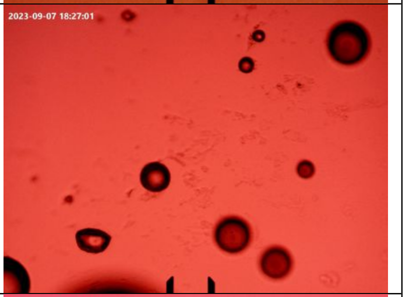
TRAS	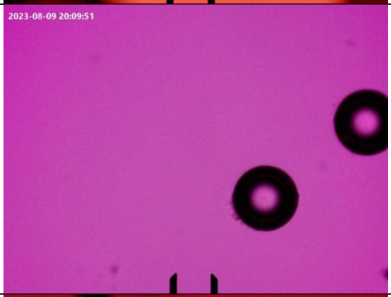	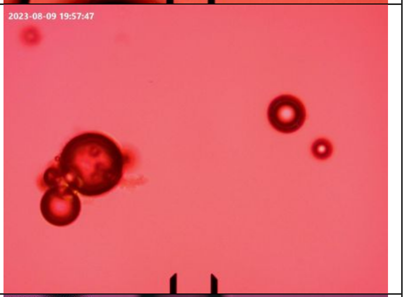
ISAT	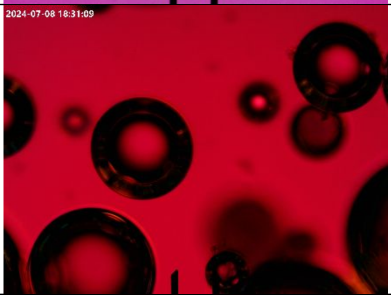	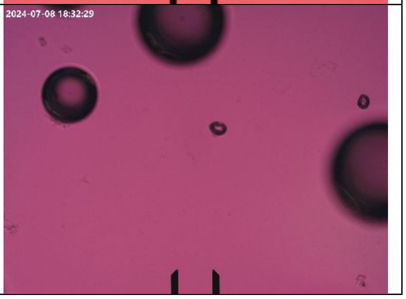

Table S3. Photos of optical observations of air bubbles in mAb solutions that were formed in real foam. Two photos per each mAb are shown.

Table S4

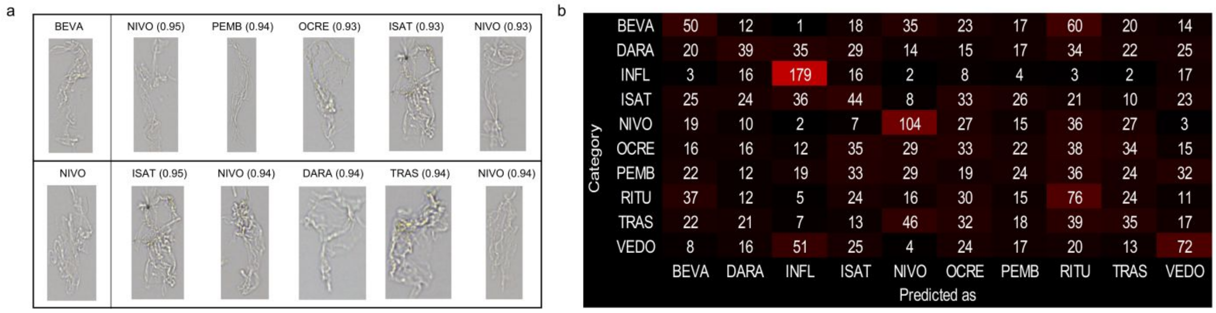

Table S4. Clustering indicates similar aggregate morphologies. (**a**) Exemplary particles from BEVA and NIVO, and the 5 most similar images determined by machine learning with origin and similarity. (**b**) Majority vote out of 5 most similar images for 250 images of each antibody subjected to clustering. The overall prediction accuracy was 0.26, indicating that only 26% of the five most similar images stem from the original mAb. Individual prediction accuracies were as follows: BEVA: 0.20, DARA: 0.16, INFL: 0.72, ISAT: 0.18, NIVO: 0.42, OCRE: 0.13, PEMB: 0.10, RITU: 0.30, TRAS: 0.14, VEDO: 0.29.

Table S5

	Surface tension σ (mN/m)				Surface pressure π (mN/m)				G 15 s (mN/m)			G 900 s (mN/m)			Elastic Modulus x Surface pressure	Foam volume (ml)	Time for blackspot formation (s)
	0 s	10 s	600 s	900 s	0 s	10 s	600 s	900 s	Elastic Mod	Loss Mod	Modulus E	Elastic Mod	Loss Mod	Modulus E			
BEVA	68.0 ± 3.3	66.7 ± 2.5	62.9 ± 0.3	62.1 ± 0.4	4.8 ± 3.3	6.1 ± 2.5	9.6 ± 0.3	10.7 ± 1.4	62 ± 0	7.7 ± 0	62 ± 0	88 ± 0.6	6 ± 0.3	88 ± 0.6	845 ± 27	0.9	50
NIVO	65.6 ± 1.2	63.7 ± 1.2	54.0 ± 0.8	53.1 ± 0.6	7.2 ± 1.2	9.1 ± 1.2	18.5 ± 0.8	19.8 ± 0.6	39 ± 3.8	5 ± 0.3	40 ± 3	43 ± 5	6 ± 0.3	44 ± 5	796 ± 99	1.5	130
RITU	69.5 ± 2.3	66.8 ± 2.9	56.1 ±1.2	55.2 ± 0.8	3.3 ± 2.3	6.0 ± 2.9	16.4 ± 1.2	17.7 ± 0.8	37 ± 2	5 ± 0.5	38 ± 2	40 ± 1.3	5 ± 0.3	41 ± 1.3	656 ± 53	0.7	22
PEMB	68.8 ± 0.4	66.9 ± 0.6	55.7 ± 0.8	54.3 ± 1.0	4.0 ± 0.4	5.9 ± 0.6	16.8 ± 0.8	18.6 ± 1.0	37 ± 2	6 ± 0.1	38 ± 2	45 ± 0.5	6 ± 0.8	46 ± 0.3	756 ± 37	1.3	65
DARA	66.4 ± 2.7	64.5 ± 2.6	55.2 ± 2.1	54.6 ± 0.9	6.4 ± 2.7	8.3 ± 2.6	17.3 ± 2.1	18.2 ± 0.9	40 ± 3	5 ± 0.6	40 ±3	39 ± 1.4	5 ± 0.7	40 ± 1.3	675 ± 85	1.1	42
INFL	71.9 ± 0.0	71.5 ± 0.3	65.0 ± 0.3	62.9 ± 0.2	0.9 ± 0.0	1.3 ± 0.3	7.5 ± 0.3	9.9 ± 0.4	56 ± 36	5 ± 1	56 ± 36	77 ± 3	6 ± 1.5	77 ± 3	578 ± 32	1.2	11
OCRE	72.1 ± 0.0	71.6 ± 0.1	65.0 ± 0.1	65.4 ± 0.2	0.7 ± 0.0	1.2 ± 0.1	6.5 ± 0.1	7.4 ± 0.2	36 ± 5	5 ± 1	37 ± 5	73 ± 6	5 ± 3	73 ± 6	475 ± 40	0.5	20
VEDO	71.5 ± 0.5	71.5 ± 0.7	65.0 ± 0.2	63.6 ± 1.1	1.3 ± 0.5	1.3 ± 0.7	7.5 ± 0.2	9.2 ± 0.7	23 ± 13	2.1 ± 1	23 ± 13	52 ± 1	4.5 ± 0.5	53 ± 1	390 ± 13	0.5	37
TRAS	71.9 ± 0.3	71.5 ± 0.2	65.0 ± 0.1	64.0 ± 0.4	0.9 ± 0.3	1.3 ± 0.2	7.5 ± 0.1	8.8 ± 0.3	19 ± 0	2.4 ± 0	19 ± 0	55 ± 1.7	4.4 ± 0.4	55 ± 1.7	413 ± 14	0.2	41
ISAT	68.9 ± 1.3	67.1 ± 1.3	55.9 ± 0.9	54.7 ± 0.4	3.9 ± 1.3	5.7 ± 1.3	16.6 ± 0.9	18.1 ± 0.5	34 ± 6	6 ± 0.1	34± 6	31 ± 4.3	4 ± 0.3	32 ± 4.3	515 ± 77	0.7	20

Table S5. Surface tension, surface pressure, elastic moduli at different timepoints, as well as foam volume and time for blackspot formation for ten mAbs. The product of the Elastic Modulus and Surface Pressure was calculated from the surface pressure at 600 s and the Elastic Modulus at 900 s. All measurements were performed at 0.45 mg/ml in acetate buffer (pH 5, with 0.9% NaCl).
